# Recruitment, follow-up and survival in an 11-country cohort study of patients at the end of life and their relatives

**DOI:** 10.1371/journal.pone.0317002

**Published:** 2025-01-09

**Authors:** Maria E. C. Schelin, Christel Hedman, Pilar Barnestein-Fonseca, Martina Egloff, John Ellershaw, Dagny Faksvåg Haugen, Claudia Fischer, Melanie Joshi, Ida J. Korfage, Urška Lunder, Stephen Mason, Judit Simon, Vilma A. Tripodoro, Berivan Yildiz, Sofia C. Zambrano, Steffen Eychmueller, Lia van Zuylen, Agnes van der Heide, Carl Johan Fürst, the iLIVE Consortium

**Affiliations:** 1 Institute for Palliative Care, Region Skåne and Department of Clinical Sciences Lund, Lund University, Lund, Sweden; 2 Department of Molecular Medicine and Surgery, Karolinska Institutet, Stockholm, Sweden; 3 R & D Department, Stockholms Sjukhem Foundation, Stockholm, Sweden; 4 Instituto CUDECA, CUDECA Foundation, Group CA15 IBIMA-Plataforma Bionand, Málaga, Spain; 5 University Center for Palliative Care, University Hospital, Inselspital Bern, Bern, Switzerland; 6 Palliative Care Unit, University of Liverpool, Liverpool, United Kingdom; 7 Regional Centre of Excellence for Palliative Care, Western Norway, Haukeland University Hospital, Bergen, Norway; 8 Department of Clinical Medicine K1, University of Bergen, Bergen, Norway; 9 Department of Health Economics, Center for Public Health, Medical University of Vienna, Vienna, Austria; 10 Department of Palliative Medicine, Faculty of Medicine and University Hospital, University of Cologne, Cologne, Germany; 11 Department of Public Health, Erasmus MC, University Medical Center Rotterdam, Rotterdam, The Netherlands; 12 University Clinic for Respiratory and Allergic Diseases Golnik, Golnik, Slovenia; 13 Instituto Pallium Latinoamérica, Buenos Aires, Argentina; 14 ATLANTES Global Observatory of Palliative Care-WHO Collaborating Centre, University of Navarra, Pamplona, Spain; 15 Institute of Social and Preventive Medicine, ISPM, University of Bern, Bern, Switzerland; 16 Department Medical Oncology, University Medical Centers Amsterdam, Amsterdam, The Netherlands; National Trauma Research Institute, AUSTRALIA

## Abstract

**Background:**

Large, international cohort studies generate high-level evidence, but are resource intense. In end-of-life care such studies are scarce. Hence, planning for future studies in terms of data on screening, recruitment, retention and survival remains a challenge.

**Objectives:**

The aim was to describe recruitment, follow-up and survival in a multinational study of patients’ and relatives’ expectations, concerns and preferences at the end of life.

**Methods:**

In this 11-country cohort study with six months follow-up patients, >18 years old, were included on the basis of an adapted “surprise question” to assess patients´ end of life status. Patients were required to be aware of their limited life expectancy. We collected patient questionnaires (baseline and 1 month), and searched medical records for the date of death. One relative per patient was invited to participate.

**Results:**

26735 patients were screened for inclusion, 3065 (11%) were found eligible and were invited to participate, 1509 chose to participate, i.e. 6% of those initially screened. A total of 699 patients (49%) participated in the 1-month follow-up, with proportions varying according to survival time, from 20% if the patient died at month 2, to 75% if the patient died at month 6. Survival time was not associated with patient gender or age, but with diagnosis, country of residence and healthcare setting.

**Conclusion:**

Approximately 20 times the desired cohort size had to be screened for eligibility. Prognostication was difficult, we noted a wide distribution of survival after inclusion. Patients’ ability to complete follow-up questionnaires declined well before death.

## Introduction

Shared decision-making between health-care professionals and the patient and family is increasingly recognized as important in healthcare in general and for patients with palliative care needs in particular [1], and although progress has been made over the last decade, there is still room for improvement [2]. Shared decision-making is closely linked to, and facilitated by, knowledge of patients´ wishes, concerns and priorities, but in end-of-life care, often too little is known about these preferences. Further, preferences may change, and evidence suggests that stability of decisions is highly dependent on the aspect of end-of-life care being discussed [3, 4]. In addition, to achieve meaningful shared decision-making in the last months of life, prognostic awareness is needed by healthcare professionals, and for patients and families. This is the background of the EU-Horizon 2020 sponsored iLIVE-study, where we aimed to assess patients’ and relatives’ expectations, concerns and preferences at two time points during the last 6 months of life. A major challenge of the study was that patients would need a certain level of prognostic awareness before they could be asked about their end-of-life experiences. It was also unclear how well the screening tool would work to identify patients and equally unknown how the care setting would affect recruitment. To the best of our knowledge, there is very little data on which to base expectations and power calculations for this population.

The aim of the current study was to provide data on recruitment, completion of follow-up questionnaires and survival of participants recruited to an international cohort study of end-of-life care, and thereby inform and facilitate future studies in this field.

## Materials and methods

The current report is based on data collected from patients and relatives in the iLIVE study [5], an 11-country cohort study investigating the end-of-life period, from the perspective of patients (baseline and 1 month follow-up questionnaires), relatives (baseline, 1 month follow-up and post-bereavement questionnaires), healthcare staff (baseline and post-death questionnaire) and with data from patients´ medical records, including date of death and/or a last follow-up date. To include a population of patients with palliative care needs and limited life expectancy we used an adapted version of the “surprise question” [6, 7], modified from 1 year to 6 months. That is, a negative answer to the question: “Would you be surprised if this patient died within the next 6 months?” would indicate that the patient was eligible for the study. If the answer to the surprise question was uncertain the Supportive and Palliative Care Indicators Tool (SPICT) [8] was used. The iLIVE study investigated several aspects of experience and care; clinical, social, and health-economic, but the primary focus was on the *expectations*, *concerns and preferences* of patients approaching the end of life. Patients from hospitals, nursing homes and palliative care services were included. For it to be ethically justifiable to ask patients these types of questions, the patients (and family, if included) had to be aware of the limited life expectancy beforehand. The additional inclusion criteria were that the patient was 18 years of age or above, cognitively capable to give informed consent, and well enough to complete the questionnaires. All patients and family members were given written information and they signed an informed consent. We aimed to recruit a total of 2000 patients. Inclusion of patients and relatives was performed between 19^th^ May 2020 and 30^th^ June 2023 and data was accessed for research purposes from medical records during 19^th^ May 2020 to 8^th^ December 2023. The actual recruitment started at different timepoints, partly due to the COVID-pandemic; Argentina 19^th^ May 2020, Spain 1^st^ September 2020, Slovenia 22^nd^ September 2020, Germany 1^st^ October 2020, Island, Norway and New Zeeland 1^st^ February 2021, Switzerland 1^st^ April 2021, the Netherlands and Sweden 15^th^ April 2021 and United Kingdom 1^st^ May 2021. All sites closed inclusion of patients 30^th^ June 2023. Part of the authors were responsible to collect data during the project and could identify individual participants during data collection. All data is thereafter pseudonymised, and only pseudonymised data is used during data analysis.

The present analysis focused on 1) the screening process, in terms of the proportions lost at the different stages of recruitment and 2) the actual survival of the patients included and the factors (age, gender, main diagnosis, care setting, country) potentially associated with survival in this study. Age and gender were patient-reported, and main diagnosis, care setting and country were reported by the healthcare staff. For the analysis of survival, the dataset was naturally restricted to the patients with a known date of death. This reduced the number of patients available for analysis to about half of the original sample, some patients lived beyond the close of the study data collection period, and some were lost to follow-up after a short period of time. Other variables had low levels of missing data (see footnote Table 1) and complete case analysis was used. The analyses performed were descriptive, with Wilcoxon ranked-sum test to investigate statistically significant differences in median survival. The aim of the analysis was not causal, rather we provide descriptive data to facilitate future studies to be sufficiently powered and in the most relevant setting.

**Table 1:** Frequency, percentages and survival (median and inter-quartal rage (IQR)) over patient characteristics for patients with known survival (N = 918).

Patient charateristics		No.	Percentage	Median survival (days)	IQR	P-value*
**Sex**						
	Male	493	54	67	27–138	
	Female	424	46	65	28–145	0.41
**Age**						
	<60	157	17	78	31–147	
	60–70	252	27	58	29–135	
	70–80	304	33	61	26–134	
	>80	205	22	73	27–152	0.37
Diagnosis*						
	Cancer	771	87	61	27–133	
	Cardiovascular disease	31	3	95	47–191	
	Neurological disease	15	2	118	147–336	
	Pulmonary disease	45	5	50	29–145	
	Frailty due to old age	10	1	67	26–118	
	Other	16	2	114	47–178	**<0.01**
**Health-care setting**						
	Hospital	556	65	54	24–131	
	Specialized PC	253	29	78	39–149	
	Nursing home	52	6	73	28–147	**<0.01**
**Country**						
** **	Argentina	164	18	70	28–168	
** **	Switzerland	79	9	69	24–140	
** **	Germany	58	6	32	13–77	
** **	Spain	123	13	81	42–166	
** **	UK	67	7	38	27–86	
	Iceland	87	9	79	34–166	
	Netherlands	118	13	46	16–106	
	Norway	76	8	69	34–117	
	New Zealand	31	3	91	47–186	
	Sweden	55	6	97	58–182	
	Slovenia	60	7	58	30–147	**<0.01**

(*of Wilcoxon rank sum test)

Missing (N): Gender (1, reported "other"), diagnosis (30), healthcare setting (57)

The iLIVE study was approved by appropriate ethical authorities in each of the countries/institutions that included patients. All analyses for the current paper were performed by SAS Statistical Software version 8.3.

## Results

**Fig 1:**
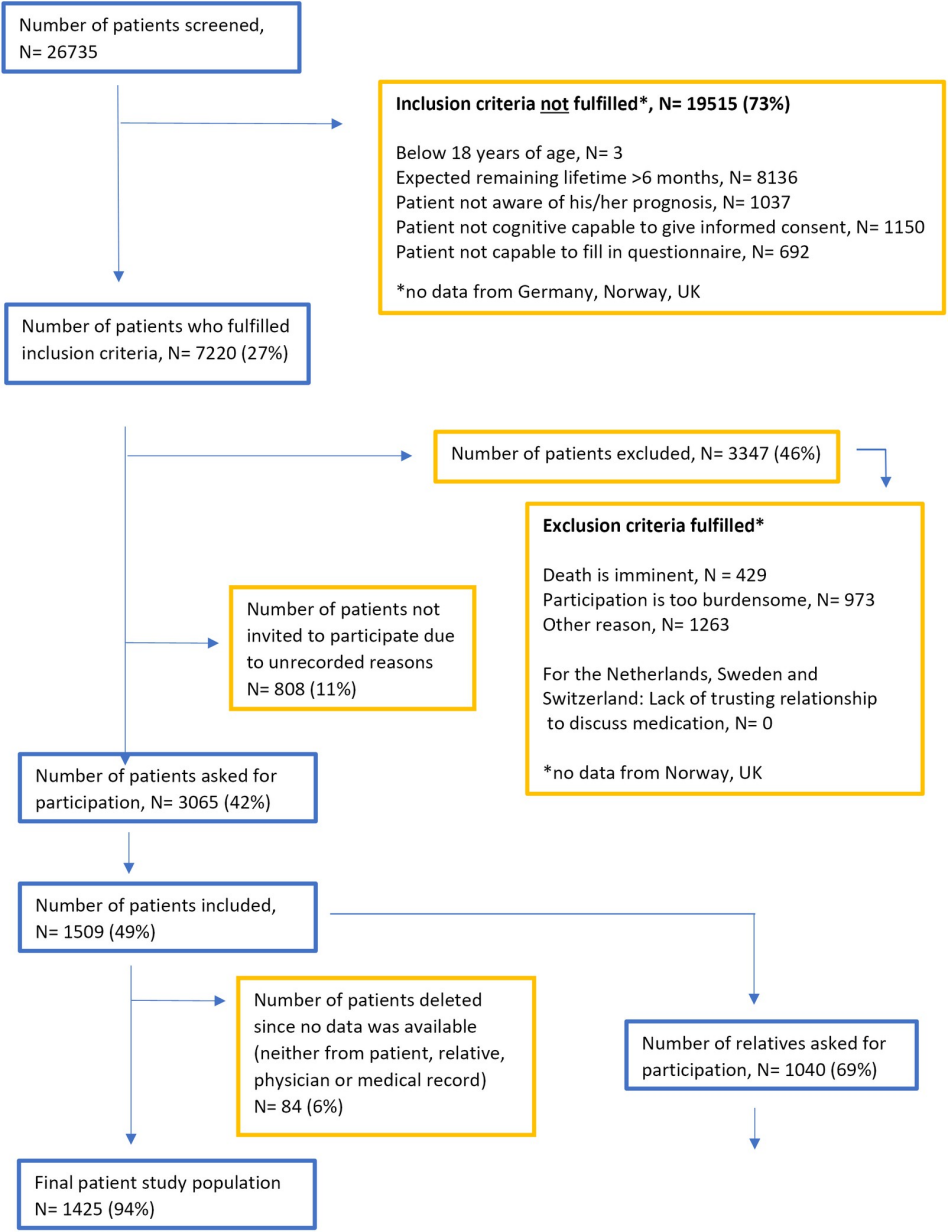
Flowchart showing inclusion of patients in the iLIVE-study.

Between 19^th^ May 2020 and 30^th^ June 2023, 26735 patients were screened for inclusion in the study, 7220 (27%) were found to fulfil the inclusion critera, and out of these 3347 were excluded due to exclusion criteria and 808 were not invited due to unrecorded reasons. Thus, 1509 patients chose to participate, corresponding to 6% of those initially screened, and 49% of those invited to participate (see Fig 1). After excluding participants without available data (neither from patient, relative, physician or medical record) 1425 patients remained. The main inclusion criterion that led to a screened patient being found ineligible was the 6-month surprise question answered affirmatively. However, three countries did not report which inclusion criterion was not met, leaving 8497 patients (32%) with an unknown reason for ineligibility. Of the included patients, 1040 (69%) both had a relative involved in their care and agreed to that the relative was invited to participate in the study, 620 (60%) of the relatives asked agreed to participate.

**Fig 2:**
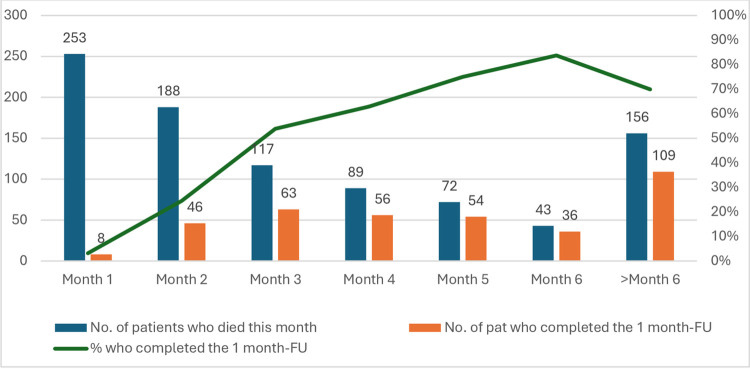
Number of patients who died and number and percentage of patients who completed the follow-up questionnaire.

A date of death was reported for 918 participants (64%). A large proportion of deaths occured in the first month after recruitment (N = 254, 28%) (Fig 2). There was a large number of patients with unknown survival status (N = 479, 33%) and a few patients who were known to have died but whose date of death was unknown (N = 27, 2%). Among patients with unknown survival status, 98 patients (26%) had a last follow-up date within the first 3 months after inclusion in the study, 59 (15%) had a last follow-up 3 to 6 months after inclusion, 177 (46%) were followed for 6 months to 1 year and 50 (13%) for longer than 1 year. Of those with a known death date, 156 patients (17%) survived the 6-month study period, and 9 patients (0,6%) had death registered without a date, but a last follow-up > 6 months after inclusion, so in total at least 392 patients (156+177+50+9, 28%) survived 6 months or longer after inclusion.

Regarding the 1-month follow-up questionnaire 699 patients (49%) completed this. In the group whose date of death was known, we observed that completion rates of the follow-up questionnaire increased with length of survival after inclusion, from 20% completion among patients who died in month 2, to 84% among patients who passed away in month 6 (Fig 1). For patients who were alive 6 months after inclusion (N = 392), the response rate to the follow-up questionnaire was 75%.

**Fig 3:**
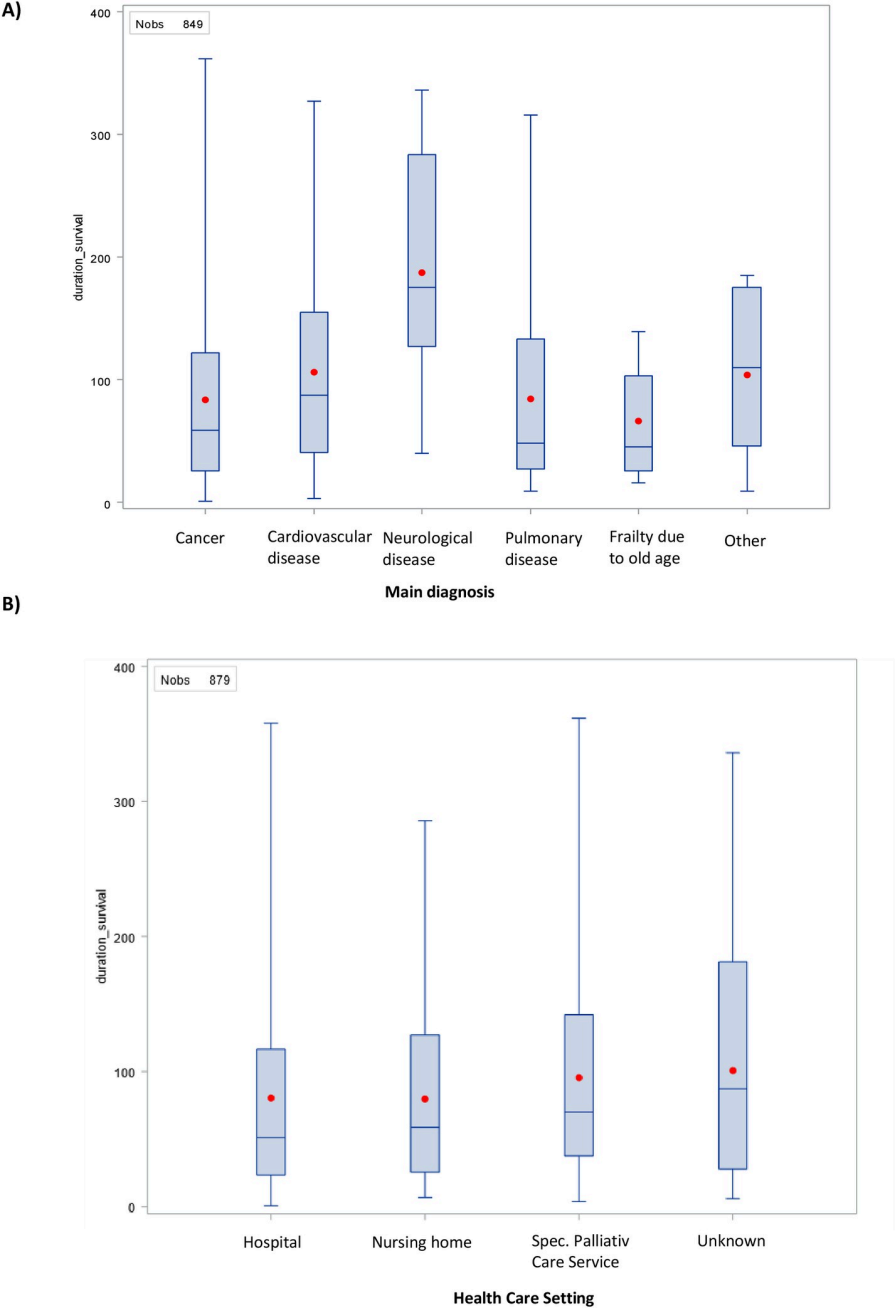
Survival by main diagnosis and healthcare setting.

We saw similar median survival across gender and age, with no statistically significant differences in these groups (p = 0.41 and p = 0.37 respectively (Table 1). However, there were statistically significant differences in survival between the three main healthcare settings where patients were recruited (hospital, nursing home and specialized palliative care service), with shorter survival in patients recruited in hospitals (median survival: 54 days), compared to specialized palliative care and nursing homes (78 and 73 days respectively). Regarding the main diagnosis, the large majority of patients (N = 771, 87%) had cancer and their median survival was 61 days. The second largest groups were patients with pulmonary disease (N = 45, 5%) with a shorter median survival of 50 days and patients with cardiovascular disease (N = 31, 3%) with a median survival of 95 days. Median survival differed statistically significantly (p<0.01) by main diagnoses. Boxplots of survival by main diagnosis and healthcare setting, restricted to the population with up to one year of survival, are presented in Fig 3. Median survival also differed significantly (p<0.01) according to which country the patients were included in.

## Discussion

In this multinational cohort study of patients at the end of life, only 11% of the screened patients were found eligible and invited to participate, but approximately half of both patients and relatives agreed to participate when invited. The willingness and/or ability to participate in the follow-up naturally decreased significantly as death approached, and we identified a decreasing trend over the whole study period of 6 months. This decline is one reason why it is important to consider the remaining life expectancy, also in a study setting. It is well-known in the field of palliative care that recruitment into research studies is difficult [9], and high attrition is to be expected, mainly due to increasing illness and death. However, our finding, that a large proportion of patients and families in end-of-life care are willing to participate in research is consistent with previous literature showing that these patients generally accept and even value research participation [10].

### Strengths and limitations

This study’s major strength is also its limitation; this is real-world data. Our hope is that reporting the challenges we experienced will help the planning of future studies. E.g., recruiting within a specific expected survival time was difficult, therefore we attempted to collect the actual date of death from medical records, even beyond the planned follow-up time of 6 months, to enable important post-hoc analyses. Unfortunately, this information was impossible to find for a large proportion (36%) of the study population, which also hampers analyses in the present report. In some cases, the patients were still alive at last follow-up in medical records and a longer follow-up period would have helped, but in 279 cases (20%) the patient was lost to follow-up within the study period or has “death” recorded but without a date. Another limitation is that, despite efforts, the screening logs, and therefore also the flowchart, were incomplete in several countries. Each country had several study sites, some countries had focused on a specific care setting, which would affect the survival time, also the screening procedures differed per site/country. It is thus likely that several mechanisms contribute to the effect of country on the difference in survival. Further, the study had a relatively low follow-up rate, especially for patients recruited close to death. The questionnaire was long (15 pages), and a shorter, more focused questionnaire would potentially have yielded higher follow-up rates. The resulting dataset is, however, one of the largest in end-of-life care, and the international setting strengthens generalizability. Our data should both provide support and encouragement to future studies that it is possible to recruit a large and meaningful sample in this highly vulnerable population, even in an international context, with different healthcare systems, cultures, end-of-life attitudes and behaviours, levels of palliative care development and communication styles.

## Conclusion

Observational studies of patients at the end of life pose several challenging research situations with very particular aspects to consider. Prognostication is notoriously difficult, but of great importance when planning even a short-term follow-up. Recruting patients requires foused effort, but once identified, patients and relatives are often willing to participate.

## Supporting information

S1 Data (XLSX)
